# Effect of 95% Ethanol as a Final Irrigant before Root Canal Obturation in Primary Teeth: An *in vitro* Study

**DOI:** 10.5005/jp-journals-10005-1327

**Published:** 2016-04-22

**Authors:** G Thiruvenkadam, Sharath Asokan, Baby John, PR Geetha Priya

**Affiliations:** 1 Senior Lecturer, Department of Pedodontics and Preventive Dentistry KSR Institute of Dental Science and Research, Tiruchengode Tamil Nadu, India; 2 Professor and Head, Department of Pedodontics and Preventive Dentistry KSR Institute of Dental Science and Research, Tiruchengode Tamil Nadu, India; 3 Principal, Department of Pediatric Dentistry, Vinayaka Missions Sankaracharyar Dental College, Salem, Tamil Nadu, India; 4 Reader, Department of Pedodontics and Preventive Dentistry KSR Institute of Dental Science and Research, Tiruchengode Tamil Nadu, India

**Keywords:** Dehydration, Ethanol, Obturation, Paper points, Primary teeth.

## Abstract

**Background:** Successful obturation in the primary teeth demands complete dryness of the root canal system.

**Aim:** The purpose of this study was to determine the effect of 95% ethanol as the final irrigant before root canal obturation in primary teeth.

**Materials and methods:** A total of 20 extracted primary mandibular canines were biomechanically prepared and pre-obturated volume of each tooth was assessed using spiral computed tomography (CT). The specimens were divided into two groups (n = 10): group 1, Metapex group; group 2, zinc oxide eugenol group. Each group was further divided randomly into two subgroups (n = 5): subgroup 1, canals were dried with 95% ethanol; subgroup 2, canals were blot dried with paper points with the last one appearing dry. All canals were obturated and the postobturated volume of each tooth was measured. The percentage of obturated volume (POV) was calculated using the formula: (postobturated volume/preobturated volume) × 100. The POV between the groups was statistically analyzed using Mann-Whitney test and Wilcoxon Signed rank test appropriately.

**Results:** Root canals that were dried with ethanol showed better obturation than using paper points alone and the difference was statistically significant in both group 1 (p < 0.001) and group 2 (p < 0.002).

**Conclusion:** Drying of the root canal system with 95% ethanol can result in better obturation in the primary teeth.

**How to cite this article:** Thiruvenkadam G, Asokan S, John B, Geetha Priya PR. Effect of 95% Ethanol as a Final Irrigant before Root Canal Obturation in Primary Teeth: An *in vitro* Study. Int J Clin Pediatr Dent 2016;9(1):21-24.

## INTRODUCTION

Preserving the integrity of primary dentition till time of exfoliation is the primary goal of pulp therapy in pediatric dentistry. It is imperative to maintain the primary dentition in healthy condition until its normal exfoliation, as it is essential for the growth and maintenance of facio-skeletal complex. The most common reason for the early loss of primary teeth is dental caries,^1^ and an alternative to avoid such loss would be endodontic treatment.

A biomechanically well-prepared root canal system along with a three-dimensional (3D) seal is the clinician’s path to success.^2^ The purpose of the obturation phase of pulp therapy is to prevent reinfection of root canals that have been cleaned, shaped and disinfected by instrumentation, irrigation and medication procedures. Successful obturation demands the use of materials and techniques that help in dense filling of the entire root canal system. It has been shown that the success of pulp therapy is dependent both on the quality of the obturation and the final restoration.^3^

Drying the root canal after instrumentation and irrigation prior to obturation is an important step in endodontic treatment. If some moisture remains in the root canal, it is impossible to obtain good obturation with the conventional obturating materials. Various materials and methods like alcohols, paper points and Luer vacuum adapter have been used to achieve proper drying of the root canal system.^4^ Alcohol has been suggested to remove the residual moisture in root canal.^5^ Literature search showed many studies evaluating the role of alcohol in permanent teeth before sealer placement. Zmener et al^6^ observed that significantly less dye leakage was present in canals that were dried with 95% ethanol; however, no significant difference was found between the canals that were dried with ethanol and paper points. Stevens et al^7^ found that a final rinse with 95% ethyl alcohol increased sealer penetration and decreased leakage in permanent root canals. Engel et al^8^ demonstrated that no significant differences were found for microleakage or sealer penetration between the groups that were finally rinsed with 70% isopropyl alcohol, sodium hypochlorite and Peridex. Paper points are still being used widely for drying the canals of primary teeth. No studies have evaluated the efficacy of alcohol in primary teeth as the final drying agent before obturation. Hence, an *in vitro* study was planned to determine the effect of 95% ethanol as a final irrigant (drying agent) before obturation in primary teeth.

## MATERIALS AND METHODS

The study design was analyzed and approved by the Institutional Review Board of KSR Institute of Dental Science and Research, Tiruchengode, Tamil Nadu, India. Primary mandibular canines extracted for space management were collected for the study. Care was taken to include only those teeth without dental caries. The final sample included 20 primary mandibular canines with little or no resorption and minimum root length of 10 mm. Collection, storage, sterilization and handling of extracted teeth followed the Occupational Safety and Health Administration guidelines and regulations. All 20 teeth were dried externally and randomly named from A to T. An access cavity was prepared in each tooth and a size 10 K-file was introduced to confirm the patency of the canal. The length was measured and the working length was derived by subtracting 1 mm from the measured length. Each tooth was enlarged to size 40 and an examiner verified the biomechanical preparation. Saline and 1% sodium hypochlorite were used as irrigants while enlarging the canals. All canals were dried with 40 size paper points. Specimens were scanned using a Light Speed VCT Scanner (GE Electricals, Milwaukee, WI, USA) and preobturated volume of each tooth was measured (X).

A total of 20 teeth were divided into two groups of 10 teeth each, that is, from A to J (group 1: Metapex) and from K to T (group 2: zinc oxide eugenol). Each group was further divided randomly into two subgroups (ethanol group and paper point group) by the secondary investigator (SI). The primary investigator (PI) was blinded of this subgroup allocation.

### Ethanol Group

The canals were irrigated with 2 ml of 95% ethanol administered with a tuberculin syringe having a 30-guage blunt-tip needle. The needle was carried to the working length and ethanol was carefully injected into the canal, while at the same time slowly moving the needle out. It was left in place for 10 seconds followed by removal of excess ethanol with a single paper point. The roots were allowed to dry for 2 minutes at 37°C to assure complete dryness.

### Paper Point Group

After irrigating with 1% sodium hypochlorite, final rinsing was done with distilled water to remove all chemicals. The canals were blot dried with paper points until complete dryness of the last point was confirmed visually.

The drying process was carried out for all the teeth by the SI and obturation was done by the PI. The teeth in group 1 were obturated with Metapex (Meta Biomed Co., Ltd., Cheongju City, Korea) and the canals were assumed to be filled when backfill occurred into the pulp chamber. A small wet cotton pellet was used to slightly compress the material inside the canal. The teeth in group 2 were obturated with zinc oxide eugenol (Septodont Healthcare India Pvt Ltd., Raigad, Maharashtra, India) using hand lentulospirals. A second spiral computed tomography (CT) was performed to assess the postobturated volume (Y) of each tooth. The percentage of obturated volume (POV) was calculated for each tooth using the formula (Y/X) × 100.

All the data were analyzed with Statistical Package for the Social Sciences (SPSS) statistical software (version 19; SPSS Inc, Chicago, IL, USA). The pre- and postobturation volume in each of the groups was compared using Mann-Whitney test. The POV between ethanol and paper point subgroups was compared using Wilcoxon signed rank test. The level of significance was set at p < 0.05.

**Table 1:** **Table 1:** Comparison of pre- and postobturated volume between the subgroups

*Group*		*Subgroup*		*N*		*Preobturated volume (X)* (mean ± SD)*		*Postobturated volume (Y)* (mean ± SD)*		*POV^†^(%)*		*p-value^‡^*	
Metapex (Group 1)		Ethanol		*5*		0.045 ± 0.009		0.042 ± 0.008		93.27		<0.001	
		Paper points		5		0.044 ± 0.005		0.037 ± 0.006		82.57			
ZnOE (Group 2)		Ethanol		5		0.042 ± 0.006		0.039 ± 0.006		93.17		<0.002	
		Paper points		5		0.041 ± 0.003		0.032 ± 0.001		79.62			

*Standard deviation; ^†^Percentage of obturated volume; ^‡^p < 0.05 considered significant

## RESULTS

**Graph 1:**
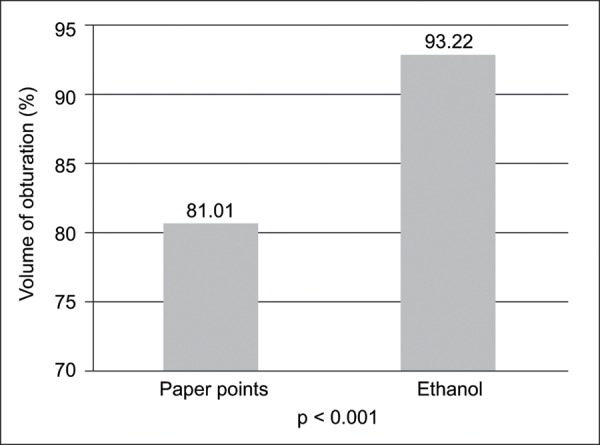
Comparison of percentage of obturated volume between paper point and ethanol groups

The mean pre- (X) and postobturation (Y) volume of root canals in each group is shown in Table 1. There was a statistically significant difference in POV (p < 0.001, p < 0.002) between ethanol and paper point subgroups in both Metapex and zinc oxide eugenol obturation respectively. Teeth irrigated with ethanol for drying showed better obturation than the teeth dried with paper points alone (Table 1). When comparing the POV between the ethanol and paper point subgroups irrespective of the obturating material, the difference was statistically highly significant (p < 0.001) in favor of ethanol subgroup (Graph 1).

## DISCUSSION

According to Epley et al,^9^ the success of a clean, well-prepared root canal system will be compromised if the root canal system is not properly obturated. Different levels of residual moisture in the root canal system have been shown to alter the sealing abilities of conventional and resin-based sealers.^6^ Thus, the quality of adhesion between root canal dentin and conventional sealers may be affected by the moisture in the root canals before obturation procedures.^10,11^

Rinsing the root canal with alcohols like ethyl alcohol before obturation has been anecdotally practiced.^7^ The basic principle is that alcohol reduces the surface tension of root canal sealers, irrigants and the root canal system.^12^ Decreasing the surface tension of a fluid or a sealer will increase the fluid flow into the dentinal tubules. Alcohol spreads into the dentinal tubules and makes the root canal dry as it evaporates. Therefore, alcohol might affect sealer penetration and seepage of the root canal filling.^12^ Tensioactive agents, such as ethanol, were found to lessen the surface tension of sodium hypochlorite and significantly improve the ability of irrigants to spread *in vitro.^12^* Stevens et al^7^ demonstrated that final rinse with 95% ethanol improved the obturation of root canals and penetration of sealers into dentinal tubules, and significantly decreased leakage. The proposed mechanism is the surfactant activity of ethanol. Dentinal dehydration may be an alternative explanation, because alcohol may not change the surface composition or its roughness.^8^ Alcohol is generally considered as a dehydrating medium. After dehydration with alcohols, dentin becomes more hydrophobic due to exposure of hydrophobic moieties and makes it more compatible with many endodontic sealers.^8^ Schafer^13^ recommended the use of approximately 3 ml of 95% ethanol per canal in order to improve the sealing ability of the root canal filling. However, the above studies were done in permanent teeth and they accessed the efficacy of sealers and their penetration into the dentinal tubules.

Traditional methods of evaluating root canal obturation in primary teeth have their own drawbacks. Intraoral radiographs give a two-dimensional (2D) interpretation only. Sectioning the root could result in loss of tooth material, which could mimic voids.^14^ Spiral CT, a noninvasive technique, gives a 3D interpretation, avoids loss of material and yields reproducible results. The specific location of voids can also be determined accurately.^15^ Hence, in this study, spiral CT was chosen as the tool for analyzing the pre- and postobturated volume.

In this study, the efficacy of two different methods of drying the primary root canals before obturation was evaluated. It was beyond the scope of the study to compare the success of obturation based on the materials used. In both the zinc oxide eugenol and Metapex groups, obturation was better with the teeth that were dried with 95% ethanol. Wong and Spencer^16^ demonstrated that dentinal tubules normally remain filled with water unless the canal is thoroughly dried. Wakabayaski et al^17^ showed that canals dried with paper points demonstrated moisture at the apical stop and in the apical third of the canal wall. Sometimes absorbent paper points cannot reach the apical end of the canal in long-curved roots. The use of ethanol helps to overcome these disadvantages.

Despite its advantages, the use of 95% ethanol in primary teeth can be questioned because of the resorbing roots and the presence of numerous accessory canals. The use of 95% ethanol is common for tissue fixation because it causes desiccation and coagulation of proteins. For tissue fixation to occur, it takes a long time and the volume of ethanol used is high. In this study, only 2 ml of the solution was used and for a very short period of time. The excess ethanol in the root canals was immediately removed with a paper point. Again, the root canal irrigant is not forced into the periapical foramen. Walters et al^18^ proved that the irrigants generally do not flow all the way to the root end in the positive pressure technique of irrigation.These facts warrant the use of ethanol in primary teeth under proper isolation and technique.

In clinical practice, a series of paper points are used to dry the canal. The use of ethanol could enhance the drying process in a cheaper and faster mode. One of the main limitations of this study was its small sample size. Considering the cost of the spiral CT and the availability of the unresorbed primary teeth, only 20 teeth were used in this study. Further studies with large sample size are warranted to improve the reliability of the results.

## CONCLUSION

On the basis of the results of this study, the following conclusions can be made:

- Primary root canals dried with 95% ethanol showed better obturation than those dried with paper points.
- The authors would like to extrapolate that it could be economical to use ethanol and a single paper point to dry the canal rather than using a series of paper points.

## References

[1] Ak G, Sepet E, Pinar A, Aren G, Turan N (2005). Reasons for early loss of primary molars. Oral Health Prev Dent.

[2] Hariharan VS, Nandlal B, Srilatha KT (2010). Efficacy of various root canal irrigants on removal of smear layer in the primary root canals after hand instrumentation: a scanning electron microscopy study. J Indian Soc Pedod Prev Dent.

[3] Ray HA, Trope M (1995). Periapical status of endodontically treated teeth in relation to the technical quality of the root filling and the coronal restoration. Int Endod J.

[4] Nagas E, Uyanik MO, Eymirli A, Cehreli ZC, Vallittu PK, Lassila LV, Durmaz V (2012). Dentin moisture conditions affect the adhesion of root canal sealers. J Endod.

[5] Spangberg L., Ingle JI, Taintor JF (1985). Intracanal medication.. Endodontics.

[6] Zmener O, Pameijer CH, Serrano SA, Vidueira M, Macchi RL (2008). Significance of moist root canal dentin with the use of methacrylate-based endodontic sealers: an in vitro coronal dye leakage study. J Endod.

[7] Stevens RW, Strother JM, McClanaban SB (2006). Leakage and sealer penetration in smear-free dentin after a final rinse with 95% ethanol. J Endod.

[8] Engel GT, Goodell GG, McClanahan SB (2005). Sealer penetration and apical microleakage in smear-free dentin after a final rinse with either 70% isopropyl alcohol or peridex. J Endod.

[9] Epley SR, Fleischman J, Hartwell G, Cicalese C (2006). Completeness of root canal obturations: Epiphany techniques versus gutta-percha techniques. J Endod.

[10] Ayad MF, Farag AM, Garcia-Godoy F (2010). Effect of lactic acid irrigant on shear bond strength of Epiphany adhesive sealer to human dentin surface. Oral Surg Oral Med Oral Pathol Oral Radiol Endod.

[11] Zicari F, Couthino E, De Munck J, Poitevin A, Scotti R, Naert I, Van Meerbeek B (2008). Bonding effectiveness and sealing ability of fiber-post bonding. Dent Mater.

[12] Cunningham WT, Cole JS, Balekjian AY (1982). Effect of alcohol on the spreading ability of NaOCl endodontic irrigant. Oral Surg Oral Med Oral Pathol.

[13] Schafer E (2007). Irrigation of the root canal. Quintessence Int.

[14] Anbu R, Nandini S, Velmurugan N (2010). Volumetric analysis of root canal fillings using spiral computed tomography: an in vitro study. Int Endod J.

[15] Reuben J, Velmurugan N, Kandaswamy D (2008). The evaluation of root canal morphology of the mandibular first molar in an Indian population using a spiral-computed tomography scan: an in vitro study. J Endod.

[16] Wong Y, Spencer P (2005). Continuity etching of an all-in-one adhesive in wet dentin tubules. J Dent Res.

[17] Wakabayaski H, Masumoto K, Tachibana H, Tuzuki N (1987). A new instrument for drying root canals. Int Endod J.

[18] Walters MJ, Baumgartner JC, Marshall JG (2002). Efficacy of irrigation with rotary instrumentation. J Endod.

